# SEOH, a novel marine-derived spirostenoid: potent broad-spectrum antimicrobial activity against multidrug-resistant aquaculture pathogens

**DOI:** 10.1007/s00253-025-13664-2

**Published:** 2025-12-19

**Authors:** Rewan Abdelaziz, Gamal EL-Didamony, Azza S. El-Demerdash, Rania A. ElDaly

**Affiliations:** 1 Department of Microbiology, Faculty of Science, Ain Shams University, Cairo, Egypt; 2 Botany and Microbiology, Faculty of Science, Zagazig University, Zagazig, Egypt; 3 Department of Biotechnology, Agricultural Research Center (ARC), Animal Health Research Institute (AHRI), Zagazig, 44516 Egypt; 4 Department of Botany and Microbiology, Faculty of Science, Arish University, Al-Arish, Egypt

**Keywords:** Antibiotic resistance, Drug discovery, Fish health, Marine natural products, *Streptomyces zaomyceticus*, Sustainable aquaculture

## Abstract

**Abstract:**

The escalating challenge of antibiotic resistance in aquaculture critically threatens global fish health and food security, underscoring an urgent need for novel antimicrobial strategies. This study explored the bioactive potential of metabolites from the marine actinomycete *Streptomyces zaomyceticus*, isolated from a deep-sea sediment sample off Sharm El-Sheikh, Egypt. Bioactivity-guided fractionation led to the isolation and structural elucidation of SPIROST-8-EN-11-ONE, 3-HYDROXY- (SEOH), identified as a novel spirostenoid. SEOH exhibited significant broad-spectrum in vitro growth inhibition against a diverse panel of aquaculture-relevant pathogens, including Gram-positive and Gram-negative bacteria, and opportunistic fungi. It demonstrated potent minimum inhibitory concentrations (MICs) ranging from 0.25 to 1.0 µg/mL, notably effective against multidrug-resistant (MDR) *Klebsiella pneumoniae* (0.25 µg/mL) and *Enterococcus faecalis* (0.5 µg/mL). Scanning electron microscopy (SEM) revealed that SEOH treatment (2× MIC) induced significant morphological alterations, including visible cell surface modifications and reduced cell numbers, in both bacterial (*E. faecalis*, *K. pneumoniae*, *P. aeruginosa*) and fungal (*C. albicans*) pathogens. Preliminary cytotoxicity assessment using the MTT assay on HepG2 cells yielded a promising IC₅₀ value of 71.76 ± 0.62 µg/ml, indicating a favorable in vitro safety profile. The novel structure of SEOH coupled with its potent, broad-spectrum in vitro antimicrobial activity against crucial aquaculture pathogens positions it as a highly promising candidate. These compelling in vitro findings strongly warrant comprehensive in vivo efficacy and safety studies to fully establish SEOH’s potential as a novel therapeutic agent or feed additive for advancing aquaculture sustainability and animal health.

**Key points:**

• *Novel Spirostenoid Discovery: SEOH, a new spirostenoid from Streptomyces zaomyceticus, was identified*

• *Potent Broad-Spectrum Activity: It shows strong inhibition against MDR aquaculture pathogens (MICs = 1.0 µg/mL)*

• *Warrants Further Study: Its promising safety profile and potency merit in vivo testing for aquaculture use*

**Supplementary Information:**

The online version contains supplementary material available at 10.1007/s00253-025-13664-2.

## Introduction

The escalating global health crisis of antimicrobial resistance (AMR), fueled by the emergence of multidrug-resistant (MDR) pathogens, necessitates the urgent discovery of novel antimicrobial agents (Akova 2016; Essawi et al. 2020; El-Demerdash et al. 2023c; Tartor et al. 2024; Al-Shemy et al. 2025). Historically, natural products, especially those originating from microorganisms, have been a prolific source of therapeutic compounds (Chopra and Dhingra 2021). Actinomycetes, a diverse group of bacteria renowned for their capacity to produce a vast array of bioactive metabolites, including numerous clinically important antibiotics such as tetracycline, erythromycin, streptomycin, and chloramphenicol, have been particularly fruitful in this regard (Chaudhary et al. 2013; Zhang et al. 2020). *Streptomyces* species*,* in particular, are prolific producers of many of these life-saving medications (Bull et al. 2005; Jose and Jha 2017; Subramani and Sipkema 2019; Donald et al. 2022). However, the widespread and often indiscriminate use of existing antibiotics has driven the evolution of AMR, underscoring the critical need to explore novel sources and innovative strategies for antimicrobial drug discovery (Abdelaziz et al. 2024; Al-Nasser et al. 2024; Mowafy et al. 2025).

The issue of antimicrobial resistance (AMR) presents a particularly acute challenge for the aquaculture industry globally. High-density farming practices, a common feature of modern aquaculture aimed at maximizing production, inherently promote the rapid transmission of bacterial pathogens among aquatic animals (Bouwmeester et al. 2021). This heightened transmission risk often leads to increased reliance on antibiotics for disease management. Furthermore, in many regions, the prophylactic use of antibiotics, intended to prevent disease outbreaks before they occur, remains a common practice, despite growing concerns about its contribution to the selection and spread of AMR (Henriksson et al. 2018). This widespread antibiotic usage in aquaculture creates a significant selective pressure, driving the emergence and proliferation of antibiotic-resistant bacteria within these environments.

Beyond the direct impact on aquaculture productivity and animal health, the spread of AMR in aquaculture has broader implications for public health. Aquaculture environments can act as reservoirs for antibiotic resistance genes, which can potentially be transferred horizontally to human pathogens (Von Wintersdorff et al. 2016; Algammal et al. 2025). This horizontal gene transfer can occur through mobile genetic elements such as plasmids and transposons, facilitating the dissemination of resistance across different bacterial species, including those that can cause infections in humans (Liu et al. 2022; El-Demerdash et al. 2023a). The interconnectedness of aquatic and terrestrial ecosystems, including the potential for resistant bacteria to enter the human food chain through aquaculture products or contaminate surrounding environments, underscores the urgent need for novel antimicrobial strategies specifically tailored to the challenges of AMR in aquaculture.

Marine environments, with their unique ecological pressures and diverse microbial communities, are recognized as a promising, yet largely untapped, source for novel bioactive compounds (Moyes et al. 2009; Ghosh et al. 2022). Among these, the marine-derived bacterium *Streptomyces zaomyceticus* has been identified as a potential producer of antimicrobial agents (Hakvåg et al. 2008). Notably, genomic analysis of *Streptomyces zaomyceticus* MP8F10, the marine isolate obtained and characterized in the study by Hakv (2009), has revealed the presence of multiple gene clusters predicted to be involved in the biosynthesis of secondary metabolites, including antibacterial compounds. These clusters, a hallmark of *Streptomyces* species renowned for their diverse chemical arsenals, often encode complex enzymatic machinery such as polyketide synthases (PKSs) and non-ribosomal peptide synthetases (NRPSs) that synthesize a wide array of clinically important antibiotics (Wang et al. 2014). The specialized ecological niches of marine *Streptomyces* often drive the production of unique secondary metabolites, distinct from their terrestrial relatives (Xu et al. 2023).

This study focuses on SPIROST-8-EN-11-ONE, 3-HYDROXY- (SEOH), a specific metabolite isolated from marine *Streptomyces*. The spirostenoid SEOH, characterized by its unique spirocyclic core and hydroxyl substitution at the C3 position, represents a novel compound. To our knowledge, its antimicrobial activity, particularly against key aquaculture pathogens, has not been previously reported. This structural novelty suggests a potentially distinct mechanism of action, offering a promising avenue to overcome prevalent antibiotic resistance mechanisms that often target conserved structural motifs (Wilson et al. 2020). Furthermore, investigating the specific biosynthetic gene cluster responsible for SEOH production in *S. zaomyceticus* could unveil novel enzymatic steps contributing to its unique structure and bioactivity. Consequently, this study demonstrated the potent antimicrobial potential of SEOH against multidrug-resistant (MDR) strains, including the bacteria *Enterococcus faecalis*, *Pseudomonas aeruginosa*, and *Klebsiella pneumoniae* and the fungus *Candida albicans*, underscoring its potential as a novel therapeutic agent in combating AMR.

## Material and methods

### Sample collection and isolation

A deep-sea sediment sample was collected from multiple bathyal zones within the Sharm El-Shaikh Sea at depths ranging from 1072 to 1167 m using a remotely operated vehicle (ROV) in September 2023 (Ramadass et al. 2010; Ramirez-Llodra et al. 2020). To minimize contamination, the collected sample was immediately transferred to a sterile plastic bag and transported to the Ain Shams University laboratory. Serial dilutions of the sediment sample were prepared in sterile saline solution and plated onto starch-nitrate agar medium supplemented with 50 mg/mL of cycloheximide and nystatin (Oxoid, Cambridge, UK) to suppress fungal growth (Antido and Climacosa 2022). While the initial isolation medium focused on fungal suppression, future studies could benefit from the inclusion of antibacterial agents such as rifampicin (30 µg/mL), to which many *Actinomycetia* exhibit resistance, to further enhance the selectivity for *Streptomyces* over other bacterial communities. The cultures were incubated at 37 °C for 5 days. A single, distinct gray colony exhibiting red pigment production was initially selected for further study. To ensure a genetically homogenous population, pure cultures were subsequently obtained through repeated streaking of this initial colony on fresh starch-nitrate agar plates until consistent colony morphology was observed.

### Morphological characterization

Morphological characterization was performed according to the International *Streptomyces* Project (ISP) criteria, including observations of mycelium morphology, colony color, substrate mycelium, melanin production, and soluble pigment production (Shirling and Gottlieb 1966).

Transmission electron microscopy (TEM) was used to examine the morphology and fine structure of the isolated strain, and comparative analysis with type strains was performed. The strain was grown on yeast extract (YE) agar at 28 °C for 21 days to obtain mature aerial mycelium and spore chains for sample preparation.

TEM samples were prepared using the spore-print technique as described by Tresner et al. (1961). Briefly, electron microscope grids covered with a collodion layer were gently pressed onto the surface of a 21-day-old culture exhibiting mature spores. Spore chains and fragments of aerial mycelium adhering to the coated surface of the grids were transferred. Grids were examined using a JEOL JEM-1600TEM transmission electron microscope operating at an accelerating voltage of 100 kV. Samples were visualized without chemical fixation or staining at required magnifications, and images were captured.

### Cultural characteristics

Growth characteristics were assessed on six standard ISP media: ISP2 (yeast extract-malt extract agar), ISP3 (oatmeal agar), ISP4 (starch nitrate agar), ISP5 (glycerol-asparagine agar), ISP6 (peptone yeast-iron agar), ISP7 (tyrosine agar), and ISP2 (yeast extract-malt extract agar) (Kumar et al. 2014). Colony morphology, including color, texture, and pigmentation, was observed on each medium. Melanin pigment production was monitored on ISP6 and ISP7 over a 14-day incubation period at 28 °C.

### Physiological and biochemical characterization

Physiological and biochemical characteristics of the isolated strain were determined following the methodologies outlined by Williams et al. (1983). This comprehensive characterization included assessing growth on various carbon and nitrogen sources, determining the temperature range for growth across 16 to 46 °C in 1 °C increments, evaluating salt tolerance in the presence of NaCl concentrations ranging from 0 to 9%, and examining pH tolerance across a range of 4 to 10 in 1 pH unit intervals. Additionally, a spectrum of biochemical tests was conducted to ascertain enzyme production (including urease and gelatinase), carbohydrate utilization profiles, and other relevant physiological characteristics.

### Molecular characterization and phylogenetic analysis

Genomic DNA was extracted from bacterial cultures grown in Tryptic Soy Broth (TSB) using the EZNA® Bacterial DNA Kit (Omega Bio-Tek, Norcross, GA, USA) following the manufacturer’s instructions. The extracted DNA was quantified using a NanoDrop 2000c spectrophotometer (Thermo Scientific, Waltham, MA, USA) by measuring the absorbance at 260 nm (A₂₆₀). DNA purity was assessed by the A₂₆₀/A₂₈₀ ratio, with values between 1.8 and 2.0 considered acceptable. The near full-length 16S rRNA gene, approximately 1500 bp in length, was amplified by PCR using the universal bacterial primers 27 F (5′-AGAGTTTGATCCTGGCTCAG-3′) and 1492R (5′-GGTTACCTTGTTACGACTT-3′) (Gillan et al. 1998). Successful amplification was confirmed through 1.2% agarose gel electrophoresis visualized under UV light. The resulting near full-length amplicon was purified using a QIAquick PCR Purification Kit (Qiagen) and sequenced by LGC Genomics GmbH (Berlin, Germany) using Sanger sequencing. The obtained 16S rRNA gene sequence was analyzed using the BLASTn algorithm against the NCBI GenBank non-redundant (nr) database for preliminary identification. For phylogenetic analysis, the sequence was aligned with reference 16S rRNA gene sequences of related *Streptomyces* species obtained from GenBank using MUSCLE Alignment Software. Phylogenetic trees were constructed using the neighbor-joining method as implemented in MEGA12 software (Kumar et al. 2024). Evolutionary distances were computed using the maximum composite likelihood method (Tamura et al. 2004). Ambiguous positions were handled using the pairwise deletion option. The robustness of the resulting topologies was assessed using 1000 bootstrap replicates (Felsenstein 1985). Type strains from several *Streptomyces* species and an *Actinomyces israelii*, *Nocardia asteroides* (GenBank: NR_118901.1), and *Micromonospora aurantiaca* (NR_117129.1) outgroup were included to root the tree. Scale bar indicates 0.02 nucleotide substitutions per sit.

### Antimicrobial activity of *Streptomyces *isolate cell-free supernatant by agar well diffusion method

The marine actinomycete *Streptomyces* isolate was cultivated in 1 L of starch-nitrate broth for 7 days at 37 °C under static conditions to produce antimicrobial metabolites. Following incubation, the culture broth was centrifuged at 5000 rpm for 10 min to separate mycelial biomass. The resulting cell-free supernatant, containing extracellular metabolites, was then filtered through a 0.45-µm sterile membrane filter to ensure sterility and remove any remaining microbial cells. This sterile cell-free supernatant was used as the test sample in the agar well diffusion assays.

The antimicrobial activity of the *Streptomyces* cell-free supernatant was evaluated using the agar well diffusion method (Economou and Gousia 2015; Hassan et al., 2019) against four multidrug-resistant (MDR) microorganisms with implications for aquaculture health: the bacterial pathogens *Enterococcus faecalis* ATCC 29212, *Pseudomonas aeruginosa* ATCC 90274, *Klebsiella pneumoniae* ATCC 13883, and the fungal pathogen *Candida albicans* ATCC 10221. All tested microorganisms were obtained from the Microbial Strain Collection of UMR-MD1, Membrane Transporters, Chimioresistance and Drug-Design, Faculty of Science, Alzahr University.

To confirm the multidrug-resistant (MDR) phenotype of the tested pathogens and establish the translational relevance of the study, a comparator-drug susceptibility assessment was performed following CLSI/EUCAST guidelines (Gaur et al. 2023). A standard panel of reference antibiotics and antifungal agents was tested against these strains. This assessment confirmed that all four test strains exhibited resistance to multiple standard therapeutic agents.

### Agar well diffusion assay for antibacterial activity

Fresh cultures of the tested bacterial pathogens were prepared to a concentration equivalent to 1.5 × 10⁸ CFU/mL (0.5 McFarland standard) in sterile saline. Sterile Mueller-Hinton agar (MHA) plates were uniformly inoculated by spreading 100 µL of the standardized bacterial suspension over the entire surface. After allowing the inoculum to dry for 5–10 min, sterile 8 mm diameter wells were aseptically punched into the agar using a sterile cork borer or pipette tip. The *Streptomyces* isolate cell-free supernatant was tested at two volumes: 100 µL and 70 µL, with each volume loaded into separate wells. Sterile broth (starch-nitrate broth) served as the negative control, and the standard antibiotic ciprofloxacin was used as the positive control in dedicated wells. The plates were then incubated at 37 °C for 24 h. Antibacterial activity was assessed by measuring the diameter of the zones of inhibition (the clear area surrounding the well indicating inhibited bacterial growth) in millimeters (mm). The zones were categorized according to Ouchari et al. (2019): very strong (> 31 mm), strong (21–30 mm), moderate (11–20 mm), and weak (≤ 10 mm).

### Agar well diffusion assay for antifungal activity

*Candida albicans* was cultured on potato dextrose agar (PDA) for 24–48 h at 28 °C to obtain fresh inoculum. A standardized fungal suspension of 1.0 × 10⁶ CFU/mL was prepared in sterile saline. Sterile PDA plates were uniformly inoculated by spreading 100 µL of the standardized fungal suspension. Sterile 8-mm-diameter wells were aseptically punched into the agar. The *Streptomyces* isolate cell-free supernatant was tested at two volumes: 100 µL and 70 µL, with each volume loaded into separate wells. Sterile potato dextrose agar (PDA) broth was used as the negative control, and nystatin served as the standard antifungal positive control. The plates were then incubated at 28 °C for 24–48 h (or until visible growth of the fungal control). Antifungal activity was assessed by measuring the diameter of the zones of inhibition in millimeters (mm) around each well.

### Metabolites extraction and analysis

#### Extraction

The *Streptomyces* isolate was cultivated in 1 L of starch-nitrate broth under static conditions at 37 °C for 7 days. Following incubation, the entire culture broth was divided into two equal portions (500 mL each). One portion was extracted with an equal volume (500 mL) of HPLC-grade ethyl acetate (Sigma-Aldrich), a solvent chosen for its ability to extract moderately polar secondary metabolites. The mixture was stirred vigorously at 200 rpm for 2 h at room temperature (approx. 20–25 °C). The organic and aqueous phases were separated using a separatory funnel after allowing the mixture to settle for at least 30 min to ensure complete phase separation. The upper ethyl acetate phase was carefully collected, dried using a Labconco CentriVap Benchtop Vacuum Concentrator at 45 °C until a completely dry residue was obtained. The resulting dry residue was then reconstituted in 5 mL of HPLC-grade methanol. The reconstituted extract was filtered through a 0.22-µm syringe filter (Millipore) and stored in sterile glass vials at −20 °C for subsequent analysis (Santos-Beneit et al. 2022; Sudha et al. 2022).

#### Initial LC–MS/MS analysis

The crude ethyl acetate extract (1 mg/mL in methanol) was subjected to initial screening by liquid chromatography-mass spectrometry/mass spectrometry (LC-MS/MS) using an Acquity ultra performance liquid chromatography (UPLC) system coupled to a ZQ4000 mass spectrometer. Chromatographic separation was performed on a Waters Bridged Ethyl Hybrid (BEH) C18 column (1.7 µm, 2.1 × 100 mm) using a linear gradient of acetonitrile (containing 0.1% TFA) in water (containing 0.1% TFA) from 10 to 100% acetonitrile over 7 min at a flow rate of 0.5 mL/min and a column temperature of 35 °C. The injection volume was 2 µL. The mass spectrometer operated in positive electrospray ionization (ESI) mode, scanning a mass range of 100–1000 m/z. For MS/MS analysis, precursor ions were fragmented using collision-induced dissociation (CID) with a collision energy of 20 eV (Annesley 2007).

#### Bioactivity-guided fractionation

The crude ethyl acetate extract was subjected to preparative high-performance liquid chromatography (HPLC) on an Agilent 1260 Infinity system equipped with a Phenomenex Luna C18 column (250 × 10 mm, 5 µm) for fractionation. The mobile phase consisted of water with 0.1% trifluoroacetic acid (TFA) (solvent A) and acetonitrile (solvent B). A linear gradient program was used at a flow rate of 5 mL/min: 0–5 min: 10% B, 5–30 min: 10–95% B, 30–35 min: 95% B, 35–40 min: 95–10% B. Fractions were collected based on UV absorbance at 210 nm using a photodiode array (PDA) detector, with one fraction collected per minute, resulting in multiple fractions. Each collected fraction was dried, reconstituted in methanol at a concentration suitable for bioactivity testing, and evaluated for antibacterial activity against *Staphylococcus aureus*. Each collected fraction was dried and subsequently reconstituted in methanol at a concentration suitable for bioactivity testing. The fractions were evaluated for antibacterial activity using the disk diffusion method. During this bioactivity-guided fractionation, *Staphylococcus aureus* ATCC 6538 was employed as an indicator strain to efficiently trace antibacterial activity. The microorganisms used for this testing were obtained from the Microbial Strain Collection of UMR-MD1, Membrane Transporters, Chimioresistance and Drug-Design, Faculty of Science, Al-Azhar University (Wang et al. 2019).

#### Characterization of the bioactive fraction

The fraction exhibiting the most potent antibacterial activity was selected for further characterization using gas chromatography-mass spectrometry (GC-MS) and nuclear magnetic resonance (NMR) spectroscopy.

##### GC-MS analysis

GC-MS analysis of the purified bioactive fraction was performed on a PerkinElmer Clarus 600 gas chromatograph coupled with a TurboMass mass spectrometer. A 2-µL sample (1 mg/mL in methanol) was injected into an Elite-5MS capillary column (30 m × 0.25 mm i.d., 0.25 µm film thickness). The oven temperature program was 40 °C for 5 min, increased to 240 °C at 5 °C/min, held at 240 °C for 2 min, increased to 300 °C at 5 °C/min, and held for 5 min. Helium was used as the carrier gas at a flow rate of 1.0 mL/min. Mass spectra were acquired in electron ionization (EI) mode in the range of 40–600 m/z (Yuan et al. 2009; Bérdy 2012).

##### NMR spectroscopy

Approximately 5 mg of the purified compound from the bioactive fraction was dissolved in 0.6 mL of deuterated chloroform (CDCl₃, Sigma-Aldrich). ^1^H and ^13^C NMR spectra were recorded on a Bruker Avance III HD 500 MHz spectrometer equipped with a 5-mm BBFO probe. Standard pulse sequences were used for data acquisition. Two-dimensional NMR experiments, including ^1^H-^1^H COSY, ^1^H-^13^C HSQC, and ^1^H-^13^C HMBC, were also performed to aid in structural elucidation. The obtained NMR data were processed using Bruker TopSpin (Qi et al. 2021).

### Cell viability assay (3-(4,5-dimethylthiazol-2-yl)−2,5-diphenyltetrazolium bromide assay)

The viability of human hepatocellular carcinoma cells (HepG2) was assessed using the 3-(4,5-dimethylthiazol-2-yl)−2,5-diphenyltetrazolium bromide (MTT) assay. We utilized the MTT assay rather than XTT because, for the HepG2 cell line and the class of compounds tested, MTT is a well-established and highly reliable method offering distinct advantages, including cost-effectiveness, simplicity of protocol, and high correlation with standard cell viability metrics in our laboratory.

Cells were seeded at a density of 1 × 10^5^ cells/ml (100 µl/well) in 96-well tissue culture plates and incubated at 37 °C with 5% CO₂ for 24 h to allow for cell attachment and monolayer formation. After 24 h, the culture medium was removed, and the cell monolayer was washed twice with phosphate-buffered saline (PBS). Serial twofold dilutions of the tested sample, which was the purified SEOH compound, were prepared in Roswell Park Memorial Institute (RPMI) medium containing 2% serum, and 100 µl of each dilution was added to the wells. Untreated cells, receiving only the RPMI medium with 2% serum, served as controls. The plate was then incubated at 37 °C with 5% CO₂ for 24 h. Subsequently, 20 µl of 5 mg/ml MTT solution (in PBS) was added to each well, followed by gentle shaking for 5 min at 150 rpm to ensure thorough mixing. The plate was then incubated at 37 °C with 5% CO₂ for 4 h to allow for the enzymatic conversion of MTT to formazan crystals. The medium was then removed, and 200 µl of dimethyl sulfoxide (DMSO) was added to each well to solubilize the formazan crystals. After 5 min of shaking at 150 rpm, the absorbance of each well was measured at 560 nm with a microplate reader, with background subtraction at 620 nm. The concentration of SEOH that inhibited cell growth by 50% (IC₅₀ value) was calculated from the dose-response curve generated by plotting SEOH concentrations against the percentage of cell viability (Iciek et al. 2012; Elashkar et al. 2024; Sewid et al. 2025). Assessing the viability of mammalian cells like HepG2 provides an initial indication of the potential toxicity of SEOH, which is crucial for evaluating its safety profile for eventual applications in aquaculture.

### Light microscopy for morphological assessment

To further investigate the cytotoxic effects of the tested compound, SEOH, on HepG2 cells, morphological changes were assessed using light microscopy. Following the same treatment conditions as described in the cell viability assay, HepG2 cells were observed at various concentrations of SEOH after 24 h of incubation. Morphological changes, including alterations in cell shape, size, and surface features, were documented to provide valuable insights into the cytotoxic mechanisms. Specifically, the presence of necrotic cells, characterized by cell swelling, chromatin flocculation, loss of nuclear basophilia, and membrane disruption, as well as apoptotic cells exhibiting distinctive features such as cell shrinkage, nuclear condensation, and fragmentation, were evaluated and recorded. Untreated cells were used as a control for normal cellular morphology.

### Determination of minimum inhibitory concentration (MIC) and minimum microbicidal concentration (MMC)

The minimum inhibitory concentration (MIC) of SEOH (SPIROST-8-EN-11-ONE, 3-HYDROXY-) was determined using the broth microdilution method (CLSI 2023; El-Demerdash et al. 2023b). Twofold serial dilutions of SEOH, ranging from 0.19 to 100 µg/mL, were prepared in sterile broth. Each well of a microtiter plate was inoculated with one of the tested microorganisms: the bacterial pathogens *Enterococcus faecalis* ATCC 29212, *Pseudomonas aeruginosa* ATCC 90274, *Klebsiella pneumoniae* ATCC 13883, or the fungal pathogen *Candida albicans* ATCC 10221, in the presence of the serially diluted SEOH.

The MIC assay included the following controls. Positive growth control wells contained sterile broth inoculated with the respective microorganism but without the addition of SEOH, confirming the viability and growth of each tested microorganism under the assay conditions. Negative growth control wells, also known as sterility control wells, contained only sterile broth without any microorganism inoculation or SEOH, ensuring the sterility of the broth and the absence of contamination.

To determine the minimum microbicidal concentration (MMC), 30 µL aliquots were taken from wells showing no visible growth in the MIC assay and subcultured onto MHA agar plates. These plates were then incubated at 37 °C for 24–48 h. The MMC was defined as the lowest concentration of SEOH that resulted in no visible growth on the agar plates after this subculturing period, indicating the concentration required to kill the microorganisms. All experiments were performed in triplicate to ensure the reliability of the results.

### Scanning electron microscopy (SEM) analysis

To investigate the morphological changes induced by SEOH, the discovered metabolite of *Streptomyces* isolate, on the tested pathogens, scanning electron microscopy (SEM) was performed (Shaaban and El-Mahdy 2018; El-Demerdash et al. 2024). The bacterial pathogens were individually cultured in fresh Mueller-Hinton (MH) broth until they reached the logarithmic growth phase. The microbial cultures were then treated with SEOH at a concentration of 2× MIC for 24 h.

Following treatment, the bacterial cells were washed three times with sterile phosphate-buffered saline (PBS; Thermo Fisher Scientific) and centrifuged at 5000 rpm for 10 min at 4 °C. The cell pellet was fixed with 2.5% glutaraldehyde (Shanghai Aladdin Biochemical Technology Co., Ltd.) at 4 °C for subsequent SEM analysis using an SU8100 scanning electron microscope (Hitachi, Ltd., Japan).

## Results

### Strain isolation and identification

A single actinomycete strain, exhibiting a distinct gray colony with red pigment production, was isolated from a deep-sea sediment sample collected from the Sharm El-Shaikh Sea. This isolate was subjected to detailed characterization, with the results illustrated in Table 1.

**Table 1:** Comparative characteristics of *Streptomyces zaomyceticus* strain SEOH and the type strain DSM 40163

Characteristic	Type strain (*S. zaomyceticus* DSM 40163)	Isolate SEOH (*Streptomyces zaomyceticus* strain SEOH)	Comparison
Aerial mycelium color	Gray to white	Gray with reddish pigment	Similar (+ red hue)
Spore chain morphology	Rectiflexibilis	Rectiflexibilis	Identical
Spore surface	Smooth	Smooth	Identical
Substrate mycelium	Yellowish	Brownish	Distinct
Aerial mycelium color	Ivory to pale yellow	Gray with reddish pigment	Distinct
Diffusible pigment on ISP-2	None (melanin negative)	Brown	Distinct
Growth temperature range	20–37 °C (optimum 28 °C)	20–40 °C (optimum 30 °C)	Slightly broader
NaCl tolerance	Up to 3%	Up to 4%	Higher tolerance
Urease	+	+	Same
Arginine dihydrolase	+	+	Same
Starch hydrolysis	+	+	Same
Hydrogen sulfide production	–	+	Different
Acetoin (Voges–Proskauer)	+	+	Same
Carbon sources utilized	Glucose, glycerol, starch	Glucose, glycerol, starch	Identical

### Characterization of Streptomyces species and antibacterial activity

#### Morphological, cultural, and biochemical characterization

**Fig. 1:**
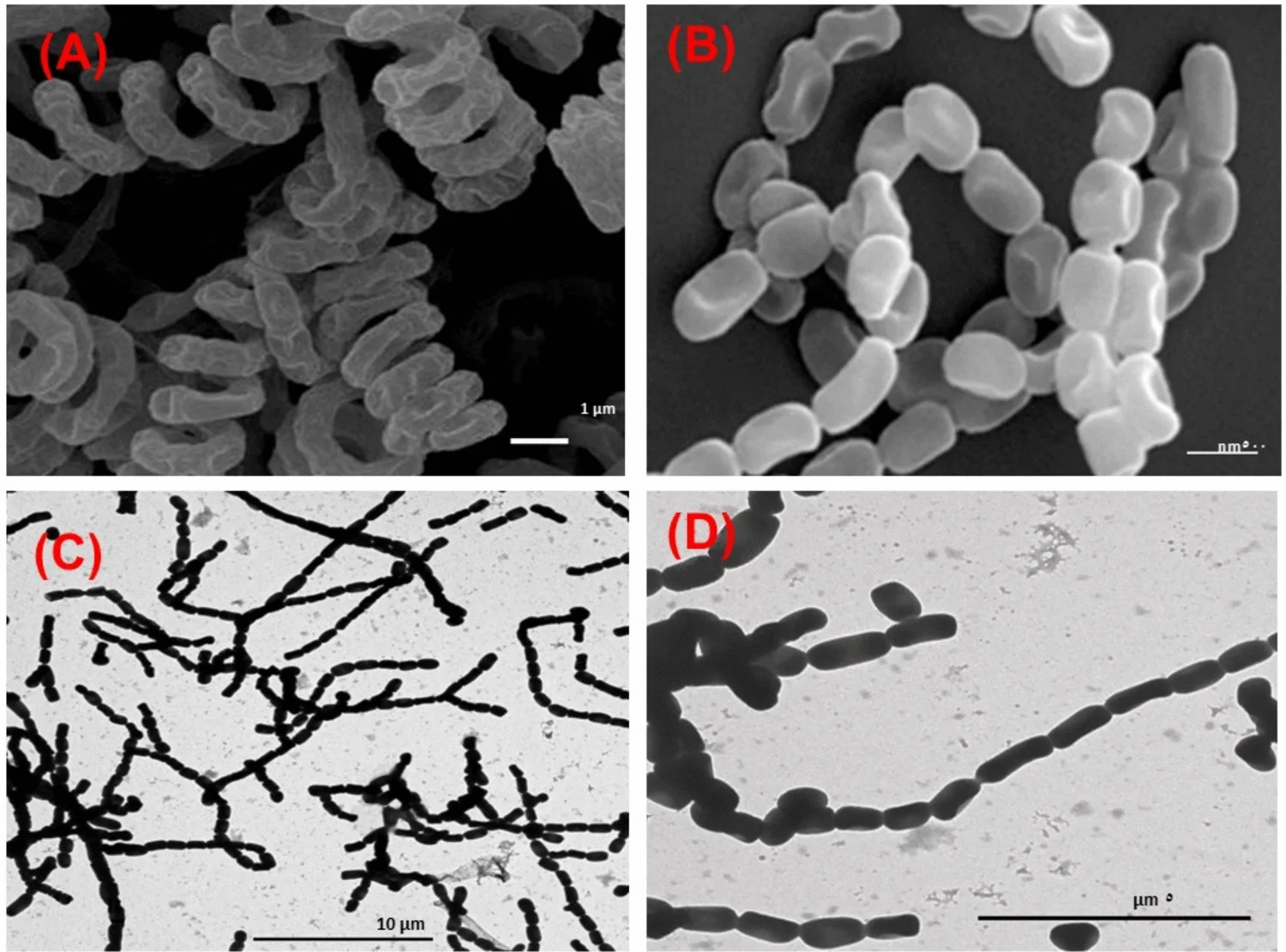
Comparative morphology and TEM ultrastructure of *Streptomyces* strains. Scanning electron micrographs (SEM) illustrate the morphology of the reference type strains: **A** *S. avermitilis* DSM 46492 (Kim and Goodfellow 2002) and **B** Strain SM01 (Maiti et al. 2020). **C**, **D** Transmission electron micrographs (TEM) of the isolated *Streptomyces zaomyceticus* strain SEOH. **C** General morphology and aerial mycelium. **D** High-magnification view showing characteristic rectiflexibilis spore chains with a smooth surface texture

Comparative microscopic analysis was performed between our isolate, *Streptomyces zaomyceticus* strain SEOH, and two reference strains: the type strain *S. avermitilis* DSM 46492 (Kim and Goodfellow 2002) and strain SM01 (Maiti et al. 2020). As shown in Fig. 1, the isolated strain SEOH exhibited rectiflexibilis spore chains with smooth spore surfaces, a morphology similar to that of strain SM01. This morphology was distinctly different from the coiled spore chains observed in *S. avermitilis* DSM 46492.

Biochemical and physiological characterization of the isolated *Streptomyces* strain (designated SEOH) revealed several key metabolic features, and a detailed comparison with the *S. zaomyceticus* Type strain DSM 40163 is summarized in Table 1.

The overall morphological, biochemical, and physiological features of strain SEOH are generally consistent with those of *Streptomyces zaomyceticus*. The strain exhibited characteristic rectiflexibilis spore chains and smooth spore surfaces. Furthermore, SEOH tested positive for common enzymatic activities, including urease, arginine dihydrolase, starch hydrolysis, and acetoin production, and demonstrated the ability to utilize glycerol, glucose, and starch as carbon sources.

#### 16S rRNA gene sequencing and phylogenetic analysis

**Fig. 2:**
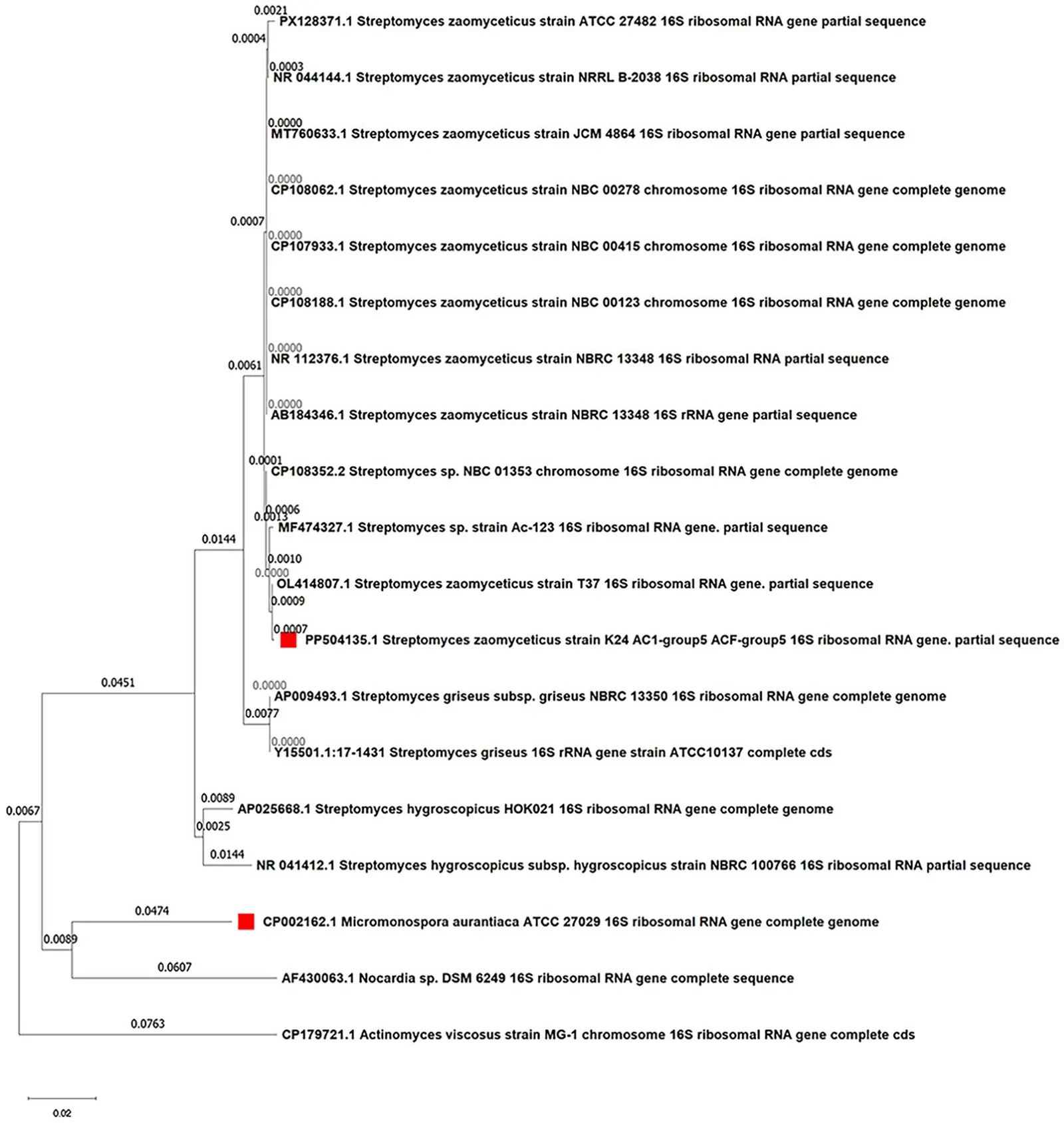
Phylogenetic tree of *Streptomyces zaomyceticus* strain K24_AC1-group5_ACF-group5. This figure depicts a phylogenetic tree constructed using the neighbor-joining method based on 16S rRNA gene sequences. The tree illustrates the phylogenetic relationship of the *S. zaomyceticus* strain K24_AC1-group5_ACF-group5 to other closely related *Streptomyces* species

For the identification of the isolated strain, the 16S rRNA gene was amplified and sequenced. Initial BLASTn analysis of the resulting sequence against the NCBI GenBank database revealed 99% sequence similarity to existing *Streptomyces zaomyceticus* strains. The determined 16S rRNA gene sequence has been deposited in the GenBank database under the accession number PP504135.1. Further phylogenetic analysis was performed using the neighbor-joining method with reference sequences of related *Streptomyces* species. The analysis showed a pairwise sequence similarity of 98.89% between the isolated strain and the *S. zaomyceticus* type strains, and an *Actinomyces israelii* outgroup was included to root the tree (Figure S1). The scale bar indicates 0.02 nucleotide substitutions per site used in the phylogenetic tree construction. The resulting phylogenetic tree (Fig. 2) consistently placed the isolated strain within the same tight cluster or subclade as the reference *S. zaomyceticus* strains. Based on the high 16S rRNA gene sequence similarity and clear phylogenetic placement, the isolated strain was confidently identified as *Streptomyces zaomyceticus*.

### Antimicrobial activity

**Fig. 3:**
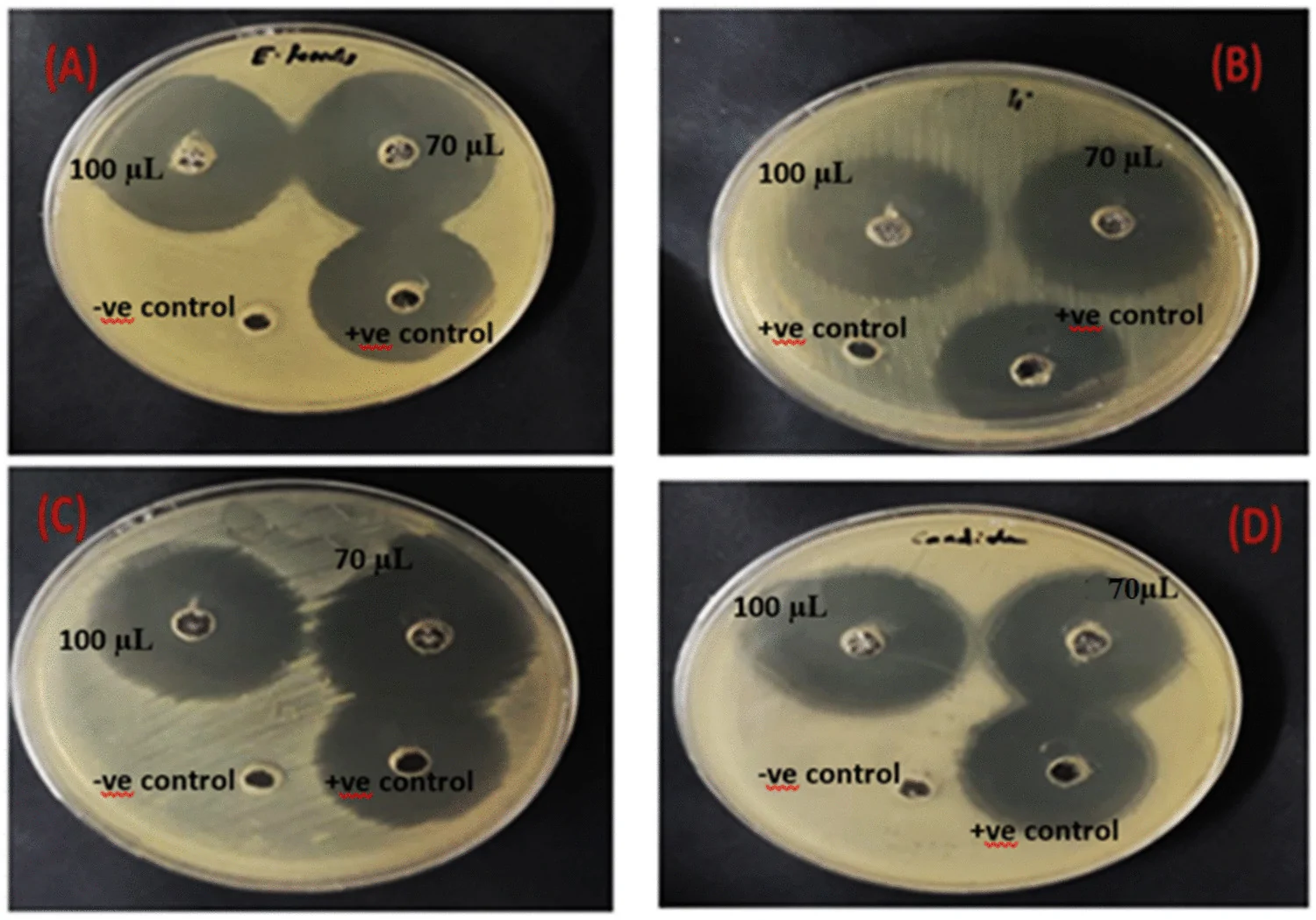
Antimicrobial spectrum of the *Streptomyces* sp. strain PP504135.1 crude extract via the agar well diffusion method. Representative images showing the growth inhibition zones around wells containing the *Streptomyces* crude extract against: **A** *Enterococcus faecalis* ATCC 29212, **B** *Pseudomonas aeruginosa* ATCC 90274, **C** *Klebsiella pneumoniae* ATCC 13883, and **D** *Candida albicans* ATCC 10221

The antimicrobial potential of the *Streptomyces* isolate was comprehensively assessed against a panel of four multidrug-resistant (MDR) pathogens relevant to aquaculture: the bacteria *Enterococcus faecalis* (ATCC 29212), *Pseudomonas aeruginosa* (ATCC 90274), *Klebsiella pneumoniae* (ATCC 13883), and the yeast *Candida albicans* (ATCC 10221). The results, as shown in Fig. 3, confirmed the isolate possesses broad-spectrum inhibitory activity against all tested microorganisms.

The isolate exhibited its highest efficacy against the Gram-positive bacterium *E. faecalis*, demonstrating the largest mean zone of inhibition at 38 ± 0.1 mm. Strong inhibitory activity was also observed against both the fungal strain *C. albicans* (34 ± 0.2 mm) and the Gram-negative bacterium *K. pneumoniae* (34 ± 0.2 mm), yielding identical mean inhibition zones. The isolate also displayed significant activity against the challenging Gram-negative bacterium *P. aeruginosa*, with a zone of inhibition measuring 31 ± 0.2 mm. Collectively, these findings underscore the potent and broad-ranging antimicrobial potential of the *Streptomyces* isolate, confirming its ability to inhibit diverse MDR pathogens across bacterial and fungal kingdoms.

### Identification and characterization of* S. zaomyceticus *metabolites

**Fig. 4:**
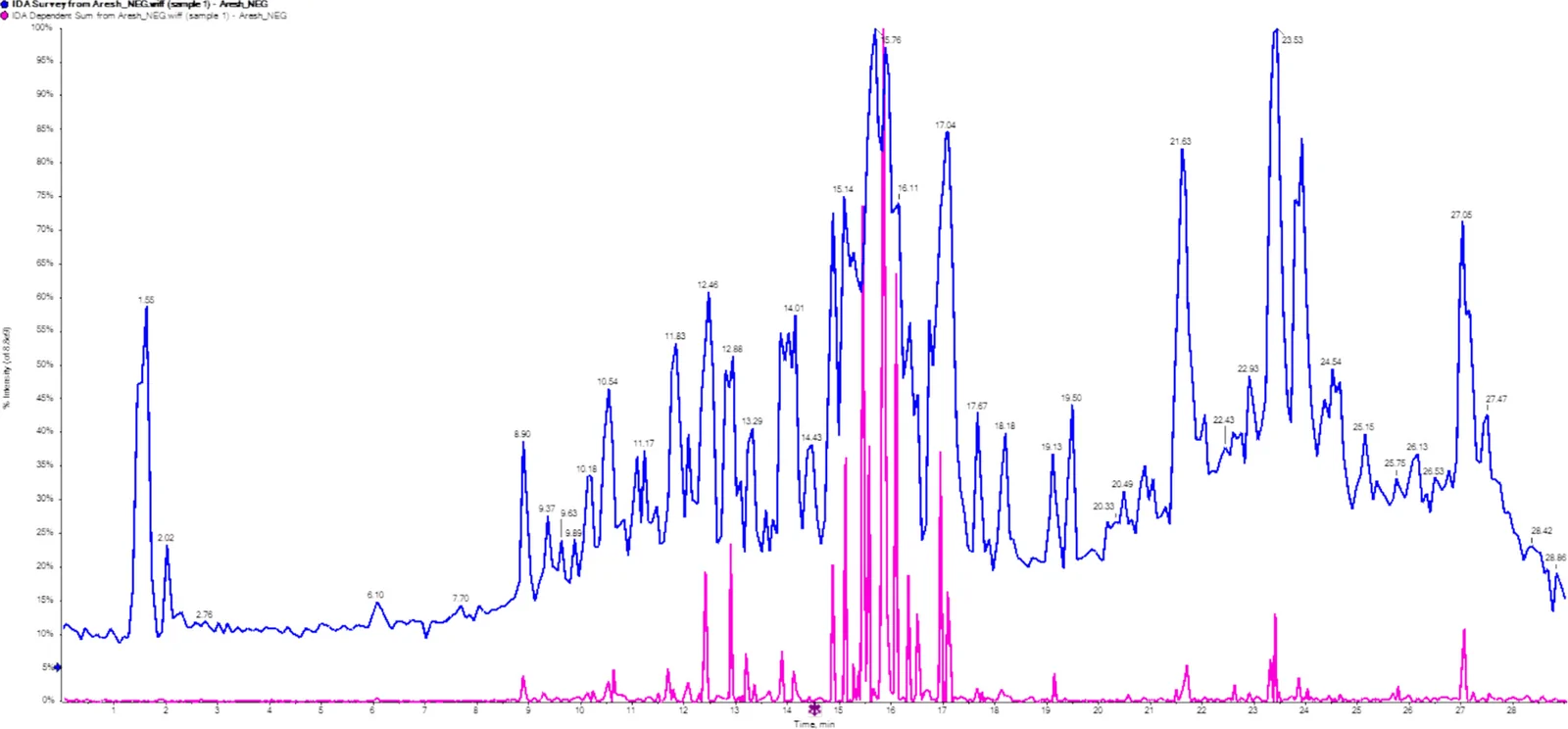
Total ion chromatogram (TIC) of*S. zaomyceticus* crude extract on day 14. The chromatogram displays major peaks of outlier compounds (numbered 1 through 7) detected in the crude extract of *Streptomyces zaomyceticus* after 14 days of cultivation. Peak labels correspond to specific compounds identified through LC–MS/MS analysis

Ethyl acetate extracts of *S. zaomyceticus* metabolites were subjected to LC-MS/MS analysis. Seven fractions were identified at retention times of 1.55, 2.10, 4.45, 13.27, 18.49, 19.56, and 27.55 min. Preliminary analysis identified these fractions as 2-DL-Glyceraldehyde 3-phosphate, L-beta-Homolysine, N-Formyl-L-Methionine, and SPIROST-8-EN-11-ONE, 3-HYDROXY- (SEOH) (Fig. 4). To guide the isolation process, the antibacterial activity of each fraction was assessed against the indicator strain, *Staphylococcus aureus* ATCC 6538, using the disk diffusion method. Fraction R5 demonstrated the most potent antibacterial activity, confirming it as the bioactive fraction for subsequent analysis.

**Fig. 5:**
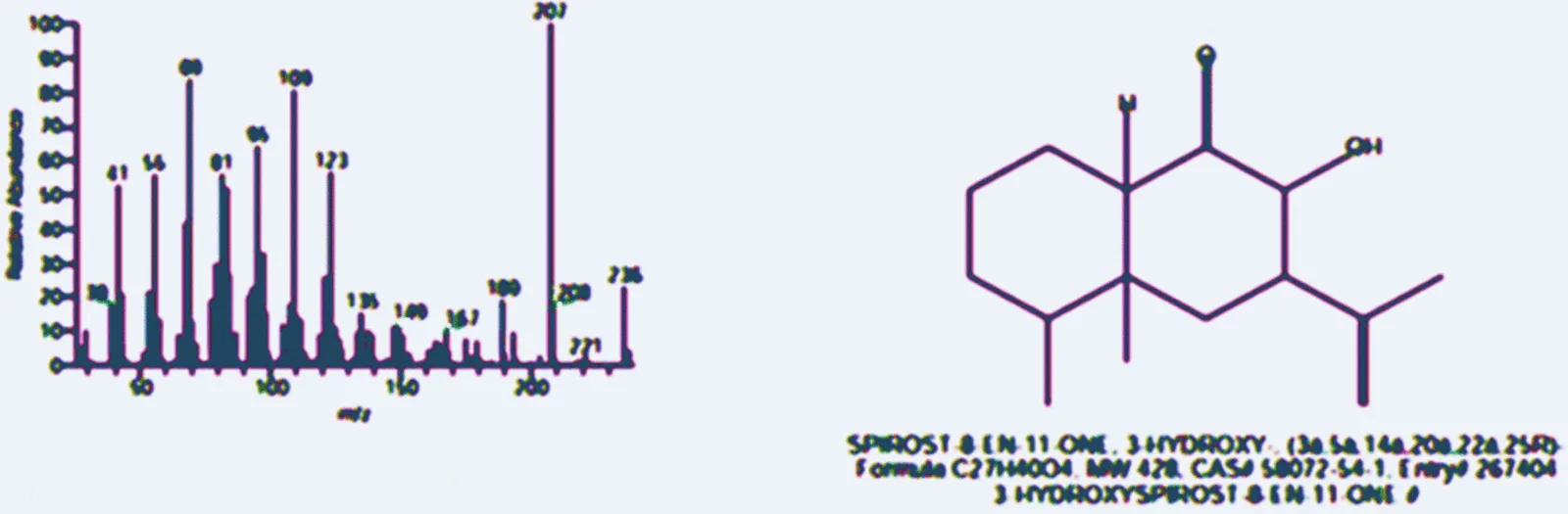
Gc mass of SPIROST-8-EN-11-ONE, 3-HYDROXY- (SEOH) with a solvent of ethyl acetate

Further characterization of the bioactive fraction (R5) was performed using GC-MS and NMR spectroscopy. GC-MS analysis revealed the presence of SPIROST-8-EN-11-ONE, 3-HYDROXY- (SEOH), a novel antibiotic metabolite. The molecular formula, molecular weight, and peak area were determined, confirming the presence of this compound. The peak area in the GC-MS chromatogram is directly proportional to the amount of the compound present in the sample (Fig. 5).

**Fig. 6:**
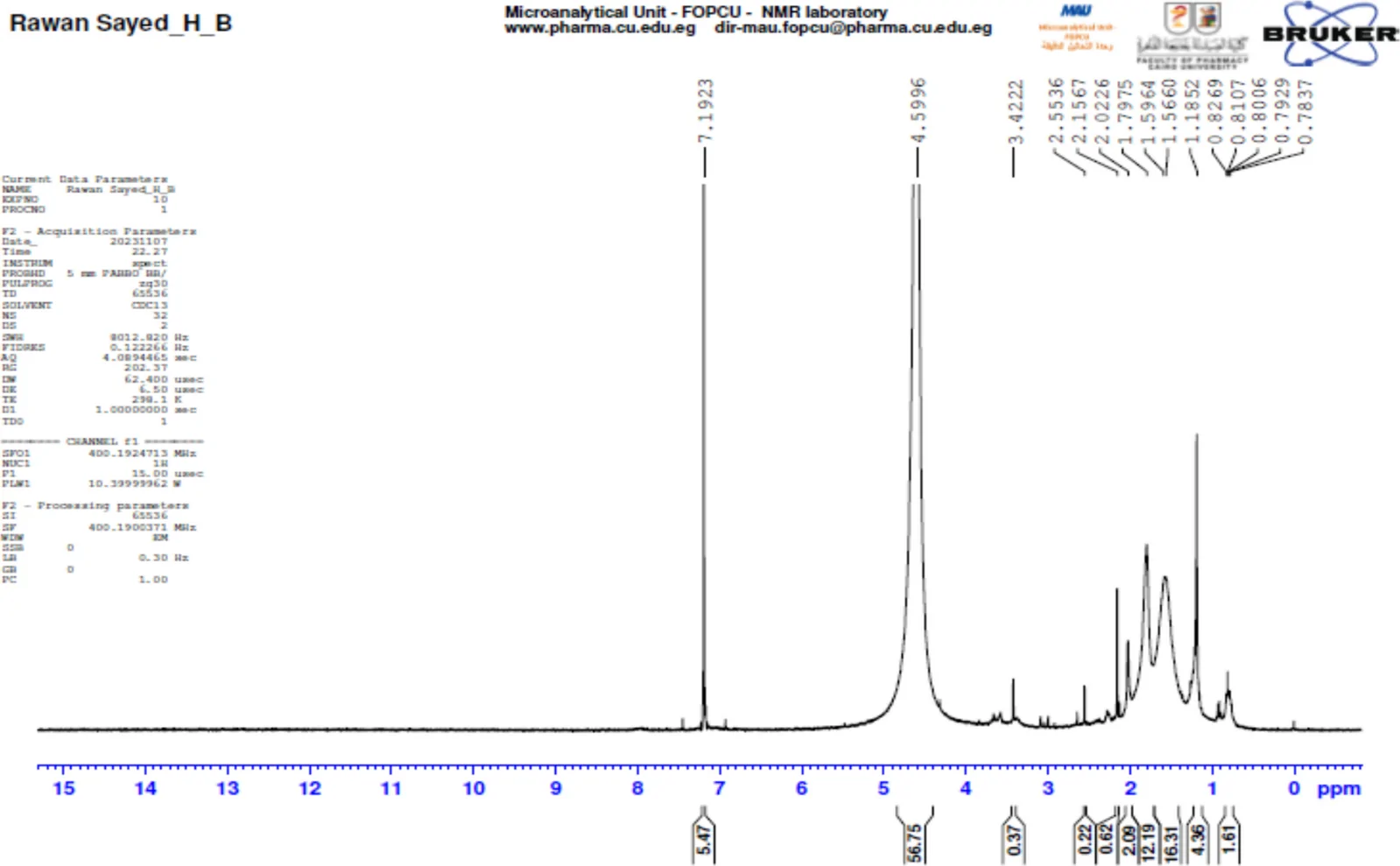
^13^C NMR spectrum of SEOH in chloroform-d. The spectrum shows characteristic peaks for the spiroketal carbon atoms, the carbonyl carbon (*δ* ~ 210 ppm), and the carbons associated with the hydroxyl and methyl groups. Chemical shifts are reported in parts per million (ppm) relative to tetramethylsilane (TMS)

 13 C NMR analysis revealed signals at *δ* 7.19 ppm (SP2 C=O), *δ* 4.59 ppm (SP2 C=N), and *δ* 3.42 ppm (aliphatic SP3 carbons), indicative of the presence of carbonyl, imine, and aliphatic functionalities. NMR analysis confirmed the presence of SPIROST-8-EN-11-ONE, 3-HYDROXY- (SEOH) as the major antimicrobial compound, as shown in Fig. 6.

### MTT assay findings

**Fig. 7:**
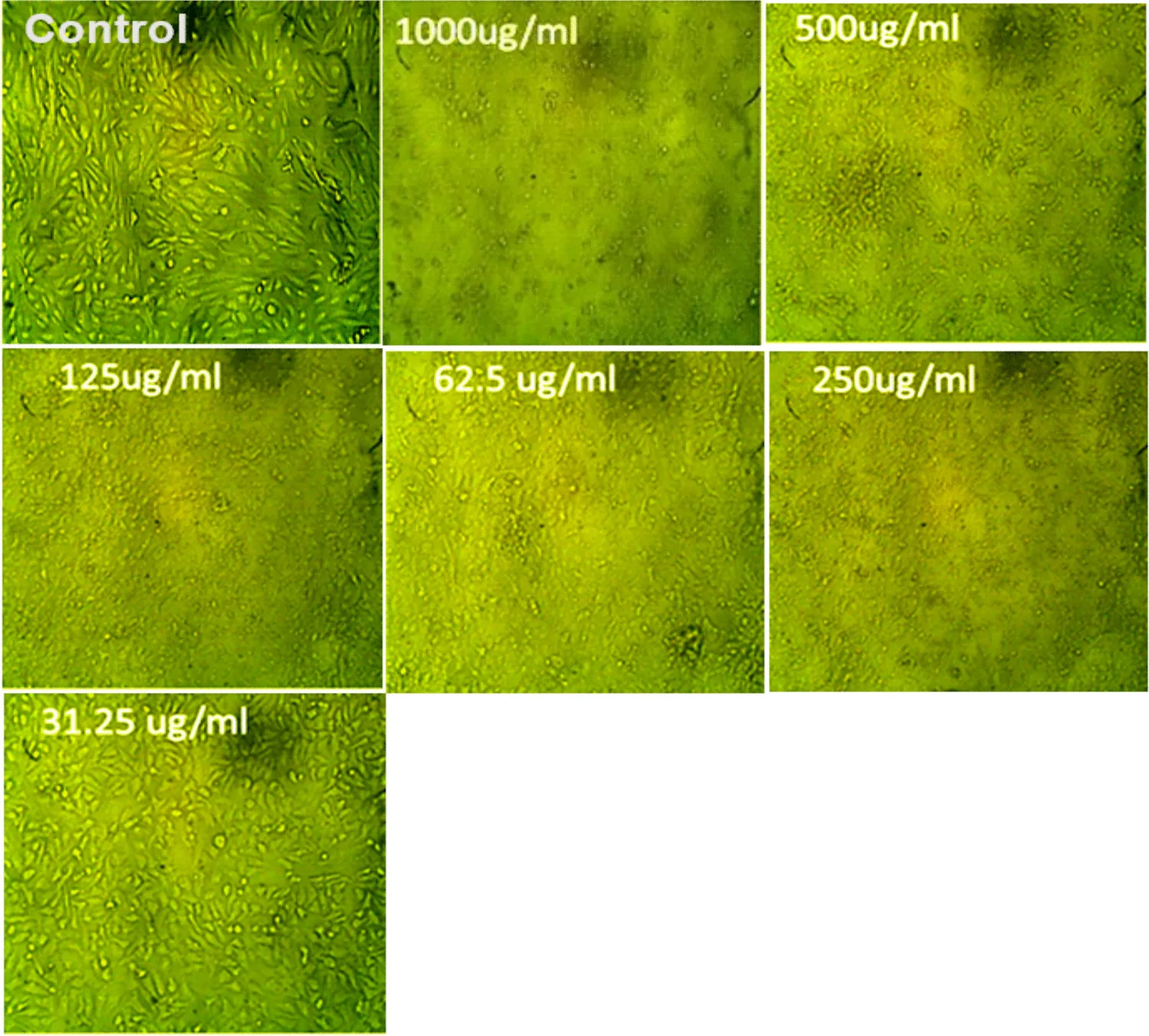
Dose-dependent cytotoxicity of SEOH on HepG2 cells. Representative images (top panels) showing HepG2 cell morphology after 24 h of treatment with decreasing concentrations of SEOH (1000 to 31.25 µg/mL). The graph (bottom panel) illustrates the dose–response relationship, showing the percentage of cell viability (blue line) and toxicity/cell death (red line) as a function of SEOH concentration. The IC₅₀ value, indicating the concentration causing 50% cell toxicity, was determined to be 71.76 ± 0.62 µg/mL

The MTT assay revealed a dose-dependent cytotoxic effect of SEOH on HepG2 cells. As the concentration of SEOH increased, a significant decrease in cell viability was observed, as evidenced by a progressive reduction in optical density values. This trend was visually corroborated by microscopic examination, which showed a clear decrease in cell density and the emergence of morphological changes, such as cell rounding and detachment, at higher concentrations. The calculated IC₅₀ value of 71.76 ± 0.62 µg/ml indicates the concentration at which SEOH inhibits cell growth by 50%. Based on this IC₅₀ value, a conservative estimate of the safe concentration for HepG2 cells is approximately 35.88 µg/ml, assuming a 50% safety margin (Table 2; Fig. 7).

**Table 2:** Cytotoxicity of SEOH on HepG2 cells

ID	Concentration (µg/mL)	O.D			Mean O.D	± SE (standard error)	Viability %	Toxicity %	IC_50 _± SD
		Replicate 1	Replicate 2	Replicate 3					
**HepG2**	--------	0.651	0.648	0.654	0.651	0.001732	100	0	ug
B	1000	0.026	0.028	0.025	0.026333	0.000882	4.045058884	95.95494112	71.76 ± 0.62
	500	0.041	0.028	0.033	0.034	0.003786	5.222734255	94.77726575	
	250	0.041	0.036	0.044	0.040333	0.002333	6.195596518	93.80440348	
	125	0.15	0.132	0.148	0.143333	0.005696	22.01740911	77.98259089	
	62.5	0.279	0.264	0.277	0.273333	0.004702	41.98668715	58.01331285	
	31.25	0.531	0.549	0.551	0.543667	0.00636	83.5125448	16.4874552

### Antibacterial activity of SEOH and morphological effects

Synthesized SEOH demonstrated significant bacteriostatic activity against all tested microorganisms: *Enterococcus faecalis* ATCC 29212, *Pseudomonas aeruginosa* ATCC 90274, *Klebsiella pneumoniae* ATCC 13883, and *Candida albicans* ATCC 10221. The minimum inhibitory concentration (MIC) and minimum microbicidal concentration (MMC) values of SEOH against these pathogens, ranging from 0.25 to 1 µg/mL, are summarized in Table 3. Beyond inhibiting growth, SEOH treatment also consistently induced distinct morphological alterations and reduced bacterial numbers across all sensitive strains, as observed by microscopic examination.

**Table 3:** Minimum inhibitory concentration (MIC) and minimum microbicidal concentration (MMC) values of SEOH against tested microorganisms

Pathogenic microorganism	MIC (µg/mL)	MMC (µg/mL)
*Enterococcus faecalis* (ATCC 29212)	0.5 ± 0.1	1.0 ± 0.1
*K. pneumoniae* (ATCC13883)	0.25 ± 0.2	0.5 ± 0.2
*Pseudomonas aeruginosa* (ATCC 90274)	1.0 ± 0.2	1.0 ± 0.1
*Candida albicans* (ATCC 10221)	0.5 ± 0.1	1.0 ± 0.1

**Fig. 8:**
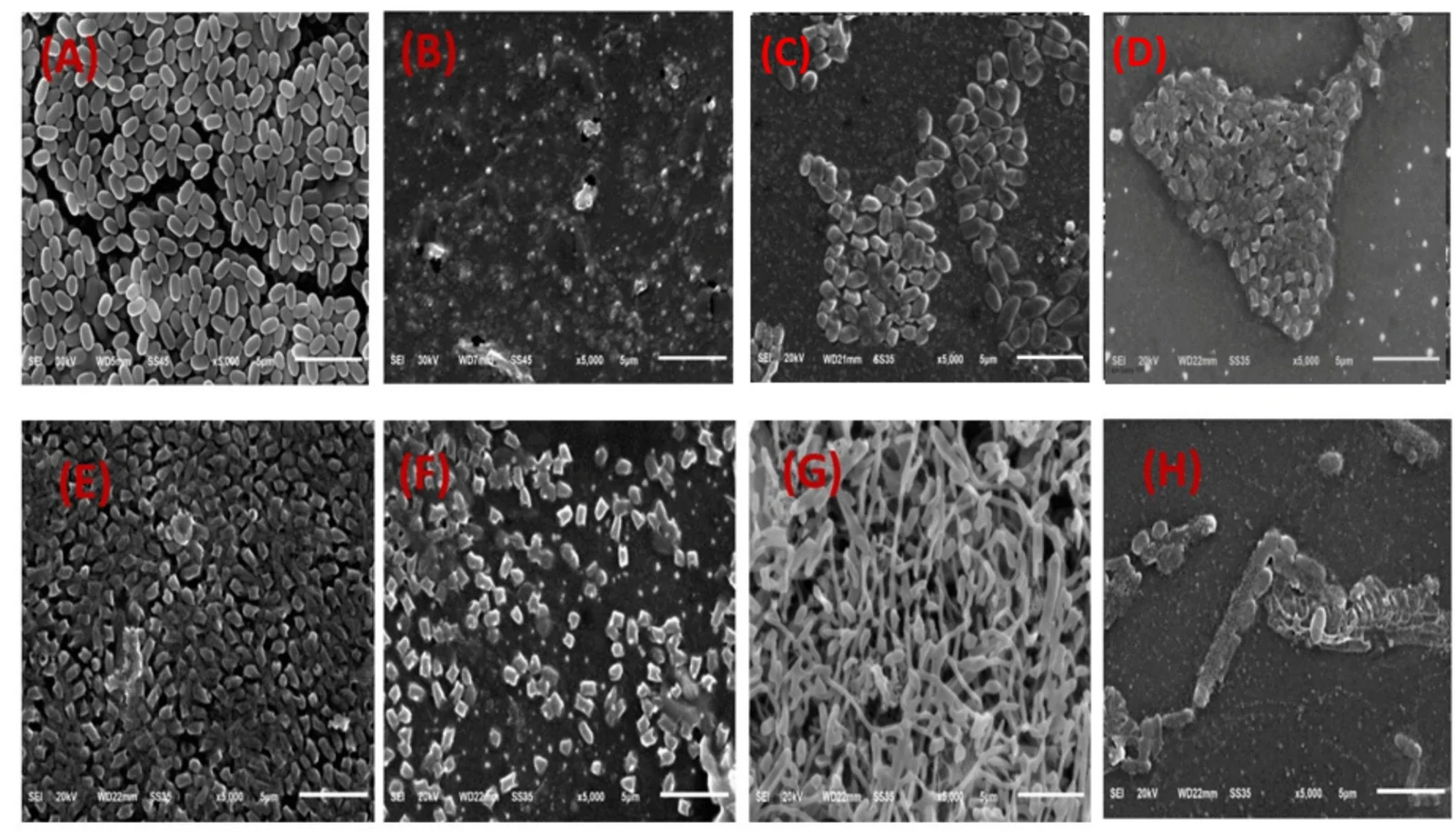
Scanning electron micrographs of *Enterococcus faecalis*, *Klebsiella pneumoniae*, *Pseudomonas aeruginosa*, and *Candida albicans* showing control groups (**A**, **C**, **E**, and **G**) and groups treated with 2 × MIC SEOH (**B**, **D**, **F**, and **H**), respectively

Specifically, for *E. faecalis*, SEOH exhibited antibacterial activity with an MIC of 0.5 µg/mL. While untreated *E. faecalis* cells displayed their characteristic non-motile, non-sporing, oval-shaped morphology typically forming chains (Fig. 8A), treatment with SEOH at 2× MIC led to significant alterations, including visible cell surface modifications and a reduction in overall bacterial numbers (Fig. 8B).

Against *K. pneumoniae*, SEOH demonstrated potent antibacterial activity with an MIC of 0.25 µg/mL. Untreated *K. pneumoniae* appeared as typical non-motile, encapsulated, rod-shaped bacteria (Fig. 8C). However, SEOH treatment resulted in significant morphological changes, notably cell surface modifications and a reduction in bacterial numbers (Fig. 8D).

For *P. aeruginosa*, SEOH’s antibacterial activity was observed at an MIC of 1.0 µg/mL. Similar to the other bacteria, while the control group exhibited the characteristic morphology of *P. aeruginosa* (Fig. 8E), SEOH treatment induced significant morphological alterations, including distinct cell surface modifications and a reduction in bacterial numbers (Fig. 8F).

### Antifungal activity and morphological effects

SEOH demonstrated antifungal activity against *Candida albicans* with an MIC of 0.5 µg/mL. Treatment with SEOH resulted in morphological alterations in *C. albicans*. The control group consisted of oval-shaped, non-motile, non-sporing cells that tended to form chains (Fig. 8G). SEOH treatment led to significant morphological changes, including cell surface modifications and a reduction in fungal numbers (Fig. 8H).

## Discussion

Our study successfully isolated and identified a marine-derived actinomycete, *Streptomyces zaomyceticus* strain SEOH, from marine sediment collected in Sharm El-Shaikh, Egypt. Phenotypic characterization, including spore morphology and biochemical assays, aligned with the typical characteristics of the *Streptomyces* genus (Baskaran et al. 2011; Jemimah Naine et al. 2012). While the overall phenotypic features were consistent with the species, the isolated strain SEOH exhibited minor but significant variations when compared to the type strain DSM 40163. These differences—particularly in unique pigment production and enhanced stress tolerance—strongly support the classification of SEOH as a novel strain of *S. zaomyceticus*. This identification was further confirmed by phylogenetic analysis based on the 16S rRNA gene sequence, which showed high sequence similarity to *S. zaomyceticus* and correctly placed the isolate within the same phylogenetic subclade.

Initial screening of the *Streptomyces* isolate using the agar well diffusion method revealed significant broad-spectrum antimicrobial activity against both Gram-positive (*Enterococcus faecalis*, *Staphylococcus aureus*) and Gram-negative (*Klebsiella pneumoniae*, *Pseudomonas aeruginosa*) bacteria, alongside moderate activity against the fungal pathogen *Candida albicans*. The observed inhibitory activity against these microorganisms is particularly relevant as *E. faecalis* and *P. aeruginosa* are recognized opportunistic pathogens causing significant economic losses in aquaculture (El-Sayed et al. 2020; Abdelsalam et al. 2021; Saad et al. 2024). Similarly, *K. pneumoniae* can contribute to environmental challenges impacting fish health (Vaneci-Silva et al. 2022; Dinev et al. 2023), and *C. albicans* is increasingly implicated in infections in stressed or immunocompromised aquaculture species (Tartor et al. 2018; Sarkar et al. 2022; Mahboub et al. 2025). This initial finding of potent antimicrobial activity against key aquaculture-relevant pathogens aligns with the well-established antimicrobial potential of *Streptomyces* species (Vijayakumar et al. 2012) and prompted the subsequent isolation and identification of the specific bioactive metabolite responsible for this promising effect.

LC-MS/MS analysis of the ethyl acetate extract led to the tentative identification of several compounds, with bioactivity-guided fractionation pinpointing fraction R5 as possessing the most potent antibacterial activity against *S. aureus*. Further structural elucidation of this fraction using GC-MS and NMR spectroscopy unequivocally identified the bioactive compound as SPIROST-8-EN-11-ONE, 3-HYDROXY- (SEOH), corroborating previous structural characterizations of this metabolite from *S. zaomyceticus* (Shandilya et al. 2017; Shankar et al. 2017).

The successful synthesis of SEOH allowed for comprehensive in vitro evaluation, revealing its potent bacteriostatic activity across all tested pathogens. As summarized by the exceptionally low MIC and MMC values (ranging from 0.25 to 1 µg/mL) presented in Table 3, SEOH demonstrated particular efficacy against the multidrug-resistant (MDR) bacterial strains *Klebsiella pneumoniae* and *Enterococcus faecalis*. While its MIC against *Pseudomonas aeruginosa* was 1.0 µg/mL, indicating strong inhibitory action, the observed antifungal activity against *Candida albicans* (MIC 0.5 µg/mL) further expanded the potential therapeutic scope of SEOH. To our knowledge, while a previous study (Bo et al. 2019) reported the isolation and characterization of SEOH from *Streptomyces*, this is the first report specifically focusing on its broad-spectrum antimicrobial activity against these clinically relevant MDR bacterial and fungal pathogens, particularly those implicated in aquaculture settings. Variations in potency levels compared to other studies could be attributed to differences in *Streptomyces* strains, extraction and purification protocols, or the specific methodologies employed for antimicrobial evaluation.

Scanning electron microscopy provided valuable insights into the mechanism of SEOH’s antimicrobial action. Treatment with SEOH at 2× MIC induced significant morphological alterations in the tested bacterial pathogens (*E. faecalis*, *K. pneumoniae*, and *P. aeruginosa*) and the fungal pathogen (*C. albicans*). The observed cell surface modifications and reduction in overall microbial numbers suggest that SEOH likely disrupts essential cellular processes, potentially targeting the cell envelope and/or interfering with cell division. These morphological changes are consistent with the effects of various known antimicrobial agents (Zhang et al. 2025). Further studies, such as membrane permeability assays and cell wall integrity assays, are warranted to elucidate the precise molecular targets of SEOH.

The MTT assay revealed a dose-dependent cytotoxic effect of SEOH on HepG2 cells, yielding an IC₅₀ value of 71.76 ± 0.62 µg/ml. This finding, while indicating potential for cellular toxicity in a mammalian liver cell line, must be carefully considered in the context of SEOH’s potent antimicrobial activity. The calculated selectivity indices (SI), which serve as an in vitro indicator of the potential therapeutic window by comparing the cytotoxic concentration to the effective antimicrobial concentrations, ranged from approximately 71.76 to 143.52 for the tested pathogens. Specifically, the highest SI was observed for *Klebsiella pneumoniae* (IC₅₀/MIC ≈ 287), followed by *Candida albicans* and *Enterococcus faecalis* (both IC₅₀/MIC ≈ 143.52). The lowest SI was noted for *Pseudomonas aeruginosa* (IC₅₀/MIC ≈ 71.76).

A selectivity index greater than 10 is generally considered promising for a potential therapeutic agent, suggesting that the compound is more toxic to the microorganism than to mammalian cells (Juretić and Simunić 2019). The relatively high SIs observed for SEOH, particularly against *K. pneumoniae*, *C. albicans*, and *E. faecalis*, suggest a favorable in vitro therapeutic potential for these pathogens.

Previous studies on structurally related spirocyclic compounds have reported varying degrees of cytotoxicity (Kim et al. 2025; Ragozzino et al. 2025). The presence and position of specific functional groups on the spirocyclic core can significantly influence the compound’s interaction with cellular targets, thereby affecting its cytotoxicity (Dhiman et al. 2022). The hydroxyl substitution at the C3 position in SEOH, along with its unique spirostenoid structure, may contribute to its specific cytotoxic profile.

The observed cytotoxicity in HepG2 cells warrants further investigation using a broader panel of mammalian cell lines, including those relevant to potential routes of administration. Furthermore, considering the potential application of SEOH in aquaculture, assessing its toxicity in relevant aquatic animal cell lines and ultimately in vivo studies in fish or shrimp models is crucial. These studies will provide a more comprehensive understanding of SEOH’s safety profile and its potential for development as a therapeutic agent in both human and animal health. The conservative estimate of a potentially safe concentration for HepG2 cells (approximately 35.88 μg/ml) based on a 50% safety margin from the IC₅₀ provides a starting point for future in vivo studies, but this needs to be validated in more complex biological systems.

The successful isolation of the spirostenoid SEOH from a marine-derived *Streptomyces zaomyceticus* strain underscores the continued potential of marine environments as a critical source of novel bioactive compounds to combat the threat of AMR. While SEOH is structurally novel and its potent bioactivity against aquaculture pathogens is newly reported, the compound has not yet been submitted for patent application. The unique structural features of SEOH, characterized by its spirocyclic core and C3 hydroxyl substitution, likely contribute to its potent antimicrobial activity and suggest a distinct mechanism of action compared to existing antibiotics, as previously hypothesized (Wilson et al. 2020).

To fully establish its therapeutic viability, essential next steps must be undertaken, including mechanistic studies (such as membrane integrity assays) and in vivo infection model validation. Additionally, further investigation into the biosynthetic gene cluster responsible for SEOH production in this marine isolate could provide valuable insights for potential heterologous expression and scaled-up production.

## Conclusion

This study has successfully unveiled SPIROST-8-EN-11-ONE, 3-HYDROXY- (SEOH), a structurally novel bioactive compound produced by the marine actinomycete *Streptomyces zaomyceticus*. The significant in vitro antibacterial activity of SEOH against key aquaculture-relevant pathogens, combined with its unique spirocyclic structure and hydroxyl substitution, strongly suggests a potentially novel mode of action. This is significant because a new mechanism of inhibiting bacterial growth could circumvent existing antibiotic resistance mechanisms that target well-established cellular processes. The reduced risk of cross-resistance would be a major advantage in combating the growing threat of antimicrobial resistance, including in aquaculture settings.

While these in vitro findings offer promising preliminary evidence for SEOH’s potential in aquaculture, we acknowledge that several critical steps are required before its consideration as a viable feed additive. Our immediate next steps will involve the following: (1) conducting in vivo efficacy trials in relevant aquaculture species to evaluate SEOH’s ability to control bacterial infections under real-world conditions; (2) performing comprehensive safety and toxicological assessments (including LD_50_/LC_50_determination in target species) to determine safe inclusion levels in animal feed and assess potential environmental impacts; (3) investigating formulation strategies to ensure stable and effective incorporation of SEOH into aquaculture feed, including evaluating pellet heating tolerance and leaching rates; (4) assessing the impact of SEOH supplementation on key growth performance metrics such as feed conversion ratio (FCR) and specific growth rate (SGR) in treated aquaculture species; and (5) crucially, elucidating the precise mode of action of SEOH through biochemical and molecular studies will be paramount. Identifying its specific bacterial target and the pathway it disrupts will not only confirm its novelty but also provide insights into potential resistance development mechanisms, guiding future strategies for its sustainable application.

These targeted validation plans are crucial in fully exploring the potential of SEOH as a sustainable and effective antimicrobial agent with eventual applications in aquaculture feed, contingent upon successful demonstration of in vivo efficacy, safety, stability, and positive impacts on growth performance.

## Supplementary Information

Below is the link to the electronic supplementary material.

ESM 1 (605 KB DOCX)

## Data Availability

All data used have been included in the manuscript and in supplementary file.
