# Supplementary material for: SEOH, a novel marine-derived spirostenoid: potent broad-spectrum antimicrobial activity against multidrug-resistant aquaculture pathogens
Source: Appl Microbiol Biotechnol. 2025 Dec 19;109:273. doi: 10.1007/s00253-025-13664-2 (PMC12718269; doi:10.1007/s00253-025-13664-2)
Supplement: Supplementary file 1 [file 253_2025_13664_MOESM1_ESM.docx]

**Figure S1.** Phylogenetic tree showing the relationship of the isolated *Streptomyces* *zaomyceticus* (PP504135.1) to related taxa based on 16S rRNA gene sequences.
